# A transgenic male-only strain of the New World screwworm for an improved control program using the sterile insect technique

**DOI:** 10.1186/s12915-016-0296-8

**Published:** 2016-08-30

**Authors:** Carolina Concha, Azhahianambi Palavesam, Felix D. Guerrero, Agustin Sagel, Fang Li, Jason A. Osborne, Yillian Hernandez, Trinidad Pardo, Gladys Quintero, Mario Vasquez, Gwen P. Keller, Pamela L. Phillips, John B. Welch, W. Owen McMillan, Steven R. Skoda, Maxwell J. Scott

**Affiliations:** 1 Department of Entomology, North Carolina State University, Campus Box 7613, Raleigh, NC 27695-7613 USA; 2 Panama-United States Commission for the Eradication and Prevention of Screwworm (COPEG), Pacora, Panama; 3 Smithsonian Tropical Research Institute, Naos Molecular Laboratory, Panama City, Panama; 4 USDA-ARS, Tick and Biting Fly Research Unit, Knipling-Bushland Livestock Insects Research Laboratory, 2700 Fredericksburg Rd., Kerrville, TX 78028 USA; 5 USDA-ARS, Screwworm Research Unit, Pacora, Panama; 6 Department of Statistics, North Carolina State University, Campus Box 8203, Raleigh, NC 27695-8203 USA; 7 USDA-APHIS-IS, Pacora, Panama; 8 USDA-ARS, Screwworm Research Unit, Knipling-Bushland Livestock Insects Research Laboratory, 2700 Fredericksburg Rd., Kerrville, TX 78028 USA; 9 USDA-APHIS, IS Action Programs, 2881 F&B Road, College Station, TX 77845 USA; 10 Present address: Department of Veterinary Parasitology, Madras Veterinary College, Tamil Nadu Veterinary and Animal Sciences University, Chennai, India

**Keywords:** New World screwworm, Sterile insect technique, tTA, Transformer, Male-only, Genetic control

## Abstract

**Background:**

The New World screwworm, *Cochliomyia hominivorax*, is a devastating pest of livestock endemic to subtropical and tropical regions of the Western hemisphere. The larvae of this species feed on the tissue of living animals, including man, and can cause death if untreated. Over 60 years ago, the sterile insect technique (SIT) was developed with the aim of eradicating this pest, initially from Florida but subsequently from all of North and Central America. From the outset it was appreciated that SIT would be more efficient if only sterile males were released in the field, but this was not possible until now.

**Results:**

Here, we report on the development and evaluation of the first sexing strains of *C. hominivorax* that produce only males when raised on diet without tetracycline. Transgenic lines have been developed that possess a tetracycline repressible female-lethal genetic system. Ten of these lines show high female lethality at the late larval/pupal stages and three of them present dominant female lethality. Most of the lines were comparable to the wild type parental strain in several fitness parameters that are relevant to mass rearing in a production facility. Further, three lines performed well in male mating success and male competition assays, suggesting they would be sexually competitive in the field. Consequently, one transgenic line has been selected by the New World Screwworm Program for evaluation under mass rearing conditions.

**Conclusions:**

We conclude that the promising characteristics of the selected sexing strains may contribute to reduce production costs for the existing eradication program and provide more efficient population suppression, which should make a genetic control program more economical in regions were *C. hominivorax* remains endemic.

**Electronic supplementary material:**

The online version of this article (doi:10.1186/s12915-016-0296-8) contains supplementary material, which is available to authorized users.

## Background

Insect pests that cause damage to agricultural crops and livestock are responsible for billions of dollars of annual losses in production to industrialized and developing countries [1, 2]. Integrated pest management strategies aim to suppress insect pest populations below levels that are harmful to the economy through a combination of the use of chemical insecticides, biological control, land use rotation, and monitoring [3]. This strategy keeps the use of insecticides to a minimum, which is desirable considering their adverse effects on the environment, where they suppress a whole range of non-target insects and, when used inadequately, cause insect pests to develop resistance, rendering them ineffective. In this scenario, a species-specific and ecologically friendly method of control is the sterile insect technique (SIT), which consists of rearing large numbers of insects, sterilizing them by irradiation and releasing them in the field, where they compete with fertile insects for mates without producing any offspring [4]. This method was first developed with the aim of suppressing populations of the New World screwworm [5, 6] and has since been used for several decades for the control of a variety of insect pests with results that range from population control to complete eradication [4].

The New World screwworm, *Cochliomyia hominivorax*, is an insect pest that parasitizes warm-blooded animals in tropical and subtropical regions of the Western hemisphere. Females lay their eggs, often near wounds, on the animal’s skin and after the larvae hatch, they feed on the animals’ live tissues, enlarging the wound, which can cause death [5]. This insect was responsible for the loss of hundreds of millions of dollars annually to the livestock industry of several countries and great efforts were made to control it [5]. During the 1950s the U.S. government began an eradication program based on SIT that succeeded in eliminating *C. hominivorax* from the whole country, and later, in collaboration with other countries, the fly was eradicated from all of North and Central America [5–7]. Currently, the Comisión Panamá–Estados Unidos para la Erradicación y Prevención del Gusano Barrenador del Ganado (COPEG) operates a biosecure facility dedicated to the mass rearing, sterilization, and dispersal of millions of sterile insects every week in the barrier zone between Panama and Colombia, preventing the re-introduction of screwworms from South America, where it still plagues all warm-blooded animals.

Although very successful, the current eradication program releases both sterile males and females into the field. The development and integration of a male-only strain of *C. hominivorax* is desirable because it could increase the efficiency of population suppression, as the sterile males would directly search for wild females without being distracted by co-released sterile females. Indeed, male-only sterile releases increased the efficiency of population suppression of the Mediterranean fruit fly in large field cages by 3- to 5-fold relative to bisexual releases [8]. Thus, a male-only strain could lead to considerable savings in production costs, as fewer insects should be needed to maintain the barrier zone. A more efficient control program increases both the potential to respond to insect outbreaks in regions that are now free of the pest and the probability that the technology will be transported into areas where the screwworm remains a hazard to humans and livestock.

Here, we report on the development of transgenic male-only strains of *C. hominivorax* suitable for introduction to a SIT program. Our transgenic lines carry a conditional female lethal trait such that, under permissive conditions, both sexes can be efficiently reared and under restrictive conditions only males are produced. We achieve this using the auto-regulated tetracycline transactivator (tTA) transgene, which is inhibited by addition of tetracycline to the diet [9]. Overexpression of tTA is lethal and interruption of the tTA gene with the sex-specific intron from the *transformer* gene assures that only females overexpress tTA protein as a consequence of sex-specific RNA splicing [10, 11]. The transgenic strains have been evaluated within the mass rearing facility for a variety of characteristics that are important for production, field performance, and risk assessment for a future open field release.

## Results

### Tetracycline-repressible female lethal transgenic lines

**Fig. 1:**
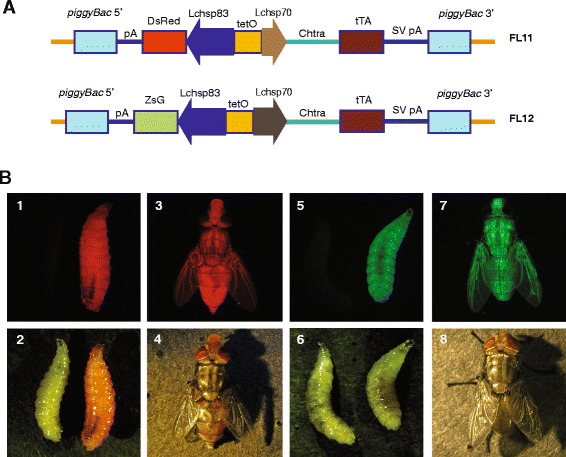
Female-specific lethal constructs. **a** The FL11 and FL12 constructs contain a conditional female-specific lethal gene and a fluorescent marker gene flanked by the ends of the *piggyBac* transposon (see text for details). **b** Fluorescent phenotypes of New World screwworm transgenic lines. Insects were observed under fluorescent (*upper panel*) and bright field illumination (*lower panel*). FL11 transgenic larvae and newly emerged adults (1 and 3, respectively) show bright *red* fluorescence in the whole body and FL12 larvae and adults (5 and 7, respectively) show strong constitutive *green* fluorescence. Fluorescence in adults cannot be seen within a few hours of eclosion due to darkening of the cuticle. Consequently, transgenic lines are identified and bred to homozygosity by screening for fluorescence at the larval stages

The FL11 and FL12 gene constructs were previously assembled containing the sex-specific intron from the *C. hominivorax tra* gene [12] inserted immediately 3’ of the tTA translation start codon (Fig. 1a) [11]. These gene constructs were used as they were available before the components of an early female lethal system had been isolated and evaluated [13]. Further, we reasoned that single insertion of a transgene could have better fitness and be easier to breed to homozygosity within the biosecure mass rearing facility. Upon induction of transcription in strains carrying the FL11/FL12 transgenes, the male tTA transcripts will retain the male-specific *tra* exon, which contains several in-frame translation stop codons. As this exon is excised in females, only the female transcript will code for the full-length tTA protein. The enhancer-promoter for the tTA gene contains 21 copies of the tTA binding site upstream of a core promoter and 5’ untranslated region from the *L. cuprina hsp70* gene [14]. The core promoter in FL12 is slightly longer than in FL11 and contains the predicted binding sites for the HSF1 transcription factor. In transgenic *L. cuprina*, both FL11 and FL12 are functional [11], but it is possible that the longer promoter in FL12 may have a higher basal activity at the temperatures that are used to rear *C. hominivorax* larvae (37–40 °C). Another difference between FL11 and FL12 is in the choice of selective marker. In FL12, the tTA gene cassette was inserted into a *piggyBac* vector that contained the ZsGreen marker driven by the strong constitutive *Lchsp83* gene promoter and in FL11 it was inserted into a *piggyBac* vector containing the *Lchsp83*-DsRed express 2 marker. Germ-line transformation of *C. hominivorax* embryos with these constructs and the *piggyBac* transposase resulted in the isolation of nine FL11 and 14 FL12 independent transgenic lines (Fig. 1b). A preliminary analysis identified four FL11 and six FL12 lines with high female lethality on standard diet without tetracycline (data not shown). These lines were reared to homozygosity and used for subsequent experiments.

**Fig. 2:**
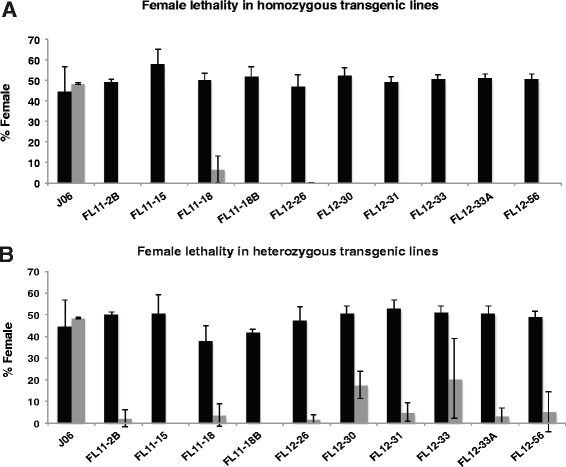
New World screwworm transgenic lines are dominant female lethal. Embryos from homozygous (**a**) or heterozygous (**b**) individuals were raised in larval diet containing (*black bars*) or without (*grey bars*) tetracycline and total number of adult males and females obtained was counted. The female lethal construct shows a high degree of dominance across all of our transgenic lines. The average percentage of total flies that were female is shown and mean ± standard deviation is expressed for three or more independent experiments

Nine homozygous transgenic lines produce only males (Fig. 2a) when reared in diet that lacks tetracycline and this effect is dominant (Fig. 2b). Indeed, the female lethal construct shows a high degree of penetrance across all 10 transgenic lines, which produce very low numbers of females when heterozygous individuals are reared in diet without tetracycline. Further, three transgenic lines, FL11-2B, FL11-15, and FL11-18B produce no heterozygous females and thus would be suitable for a fertile-release program (Fig. 2b). In these assays, flies were counted in hundreds and with typically six or seven replicates for each transgenic line (Additional files 1 and 2). All lines showed a significant decrease in female viability on diet without tetracycline (Fisher’s exact test, *P* < 0.002). A dose response assay was performed to evaluate the minimal concentration of tetracycline required to rear both males and females under permissive conditions (Additional file 3A). At 150 and 100 μg/mL, females and males were equally viable. However, at lower levels of 10 and 3 μg/mL tetracycline, female viability decreased significantly (*P* < 0.01), with few females surviving on diet with 3 μg/mL tetracycline. An alternative larval diet for screwworms is based on soy protein instead of fishmeal protein, which has the benefit of being more reproducible from batch to batch, more economic, and easier to obtain [15]. A tetracycline dose response assay is presented also for soy meal diet with similar results, observing a significant decrease in female viability on diet that contained tetracycline at 0, 3, or 10 μg/mL (*P* < 0.01) (Additional file 3B).

### General fitness of transgenic sexing strains

**Fig. 3:**
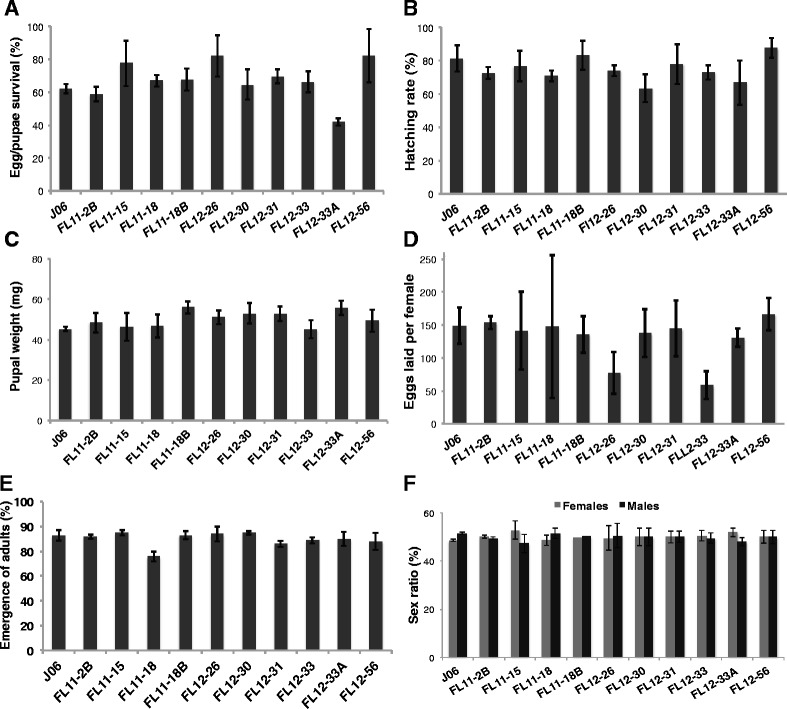
Fitness parameters of transgenic New World screwworm lines. Transgenic lines raised in diet containing tetracycline were evaluated for biological parameters important for mass rearing in a production facility: **a** percentage of embryos that develop into pupae, **b** percentage of first instars that hatch from embryos, **c** average weight of the pupae, **d** average number of eggs laid per female, **e** percentage of adults that emerge from pupae, and **f** sex ratio of emerged adults. Each experiment was performed three times for each transgenic line. Mean ± standard deviation are shown

As this study was performed within the New World screwworm biosecurity facility in Panama, all the transgenic lines were evaluated for parameters that impact mass rearing of a potential strain. In the majority of the transgenic lines, we observed a high level of productivity relative to the control strain (J06) when insects were reared in diet containing tetracycline (Fig. 3a–f). For egg/pupae survival (Fig. 3a), hatching rate (Fig. 3b), adult emergence (Fig. 3e), and sex ratio (Fig. 3f) there were no significant differences between any of the transgenic lines and the control strain J06. Lines FL11-18B and FL12-33A produced pupae that were significantly heavier than J06 (Fig. 3c, *P* < 0.05, linear model, pairwise comparisons). Line FL12-33 produced significantly fewer eggs per female than J06 (Fig. 3d, *P* < 0.05). The fact that the sex ratios of the emerged adults did not differ significantly from those of the parental J06 strain (Fig. 3f), indicates the addition of tetracycline to the diet is permissive for rearing both sexes. Female lethality in non-permissive conditions was at the late larval/pupal stage; as in most lines, only half of the number of pupae emerged as adults and all of them were male (Additional file 1). In FL11-18B, FL12-26, and FL12-56 some female lethality occurs earlier, as 67–69 % of pupae emerged as an adult all male population.

### Male mating success and male sexual competition

**Fig. 4:**
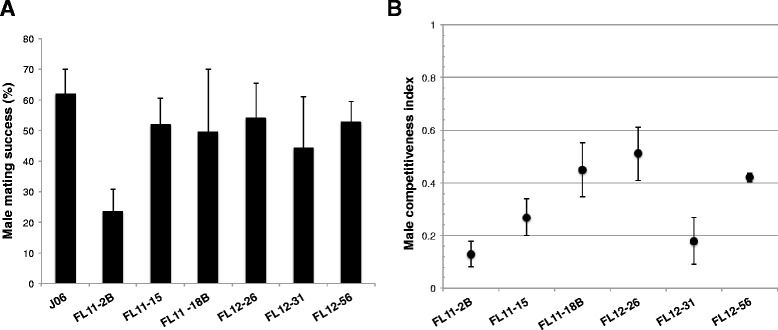
Male mating success and male sexual competitiveness. The mating success of homozygous transgenic males and that of parental J06 males (**a**) was evaluated in experiments with 5 males and 15 females allowed to mate for 8 h. The percentage of females that mated was determined by examining dissected spermatecae for the presence of sperm. The sexual performance of transgenic males was evaluated in competition experiments (**b**) containing equal numbers of mature transgenic males, wild type males, and wild type females (10:10:10) in a cage for 20 h. The offspring of individual females was examined with a fluorescence microscope to determine paternity as fluorescent larvae are obtained from females mating to transgenic males and non-fluorescent larvae resulted from mating to J06 males. The mean male competitiveness index (MCI) ± standard deviation are shown for three independent experiments. An MCI of 0.5 indicates that the transgenic strain males are equally competitive with the control strain

The transgenic lines with production and mass rearing parameters comparable to J06 were then evaluated for male mating success and sexual competitiveness in controlled mating arenas. The mating success of transgenic males was evaluated by comparing the number of females mated to males from each transgenic line and to the J06 wild type strain within a given amount of time. Generally, mating success of males was high across transgenic lines and similar to the level of success observed in the J06 strain (*P* > 0.05, pairwise comparisons, differences of line least squares means with a generalized linear model). Males from the FL11-15, FL12-26, and FL12-56 transgenic lines were the most successful in mating with females, with over 50 % of the females being inseminated within 8 hours in laboratory conditions (Fig. 4a). For all the experiments, non-irradiated males reared in diet without tetracycline were used, as we operated under the assumption that the performance of non-irradiated males predicts the performance of irradiated males, that is, that irradiation will not differentially affect wild type and transgenic males.

Focusing on the six transgenic lines that performed best in mating success assays, we studied male sexual competitiveness by presenting females with equal numbers of transgenic and J06 males, and assessing the paternity of the progeny after allowing the insects to mate for a limited period of time. Male sexual competitiveness was quantified by calculating the male competitiveness index (MCI), where an index of 0.5 indicates that transgenic and wild type males are equally competitive. We observed transgenic males from several lines competed equally well for matings in direct mating trials with the J06 strain (Fig. 4b). Males from three lines in particular, FL12-26, FL11-18B, and FL12-56, competed well against J06 males for matings with J06 females. Line FL11-15, in contrast, was significantly less competitive than J06 but only marginally so (*P* = 0.035), whereas FL12-31 and FL11-2B were out-competed for mates by J06 (*P* = 0.001, comparing observed frequency to equally competitive value of 50 % using a generalized linear model).

### Preliminary risk analysis

**Fig. 5:**
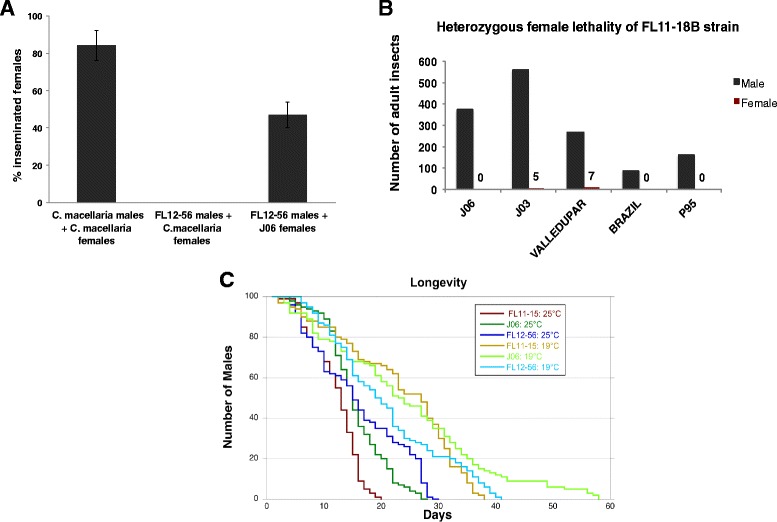
Risk analysis studies to evaluate the use of transgenic males for SIT. **a** To test the potential for outcrossing to a related species, the percentage of inseminated females is shown after mating *C. macellaria* or FL12-56 males with *C. macellaria* females. As a control, FL12-56 males were also mated with the parental J06 females. At least six replicate experiments were performed. **b** The penetrance of the female lethal construct was evaluated in different genetic backgrounds. The number of males (*black bar*) and females (*red bar*) that developed from crosses of FL11-18B males with females from the indicated wild type strains are shown. **c** Study of longevity of the sexing strains at standard and cooler temperatures. Longevity at 25 °C (*dark curves*) and 19 °C (*light curves*) of males from the J06 parental strain, the FL12-56 and FL11-15 transgenic lines. Two replicates of longevity tests with 50 males were combined and Kaplan–Meier survival curves were estimated for each combination of temperature and strain

For any planned transgenic release, the potential for outcrossing to closely related species in the field is a concern. Within Panama, *Cochliomyia macellaria* is the most closely related species of Calliphorid. This species is broadly sympatric with *C. hominivorax* across Central and South America [16, 17]. Although the two species have not been reported to hybridize in the wild and previous studies showed that they mate very rarely in caged settings, producing no hybrid offspring [18, 19], we studied the potential for transgenic *C. hominivorax* males to mate with wild type *C. macellaria* females in the laboratory. For this study, we selected one of the best performing transgenic strains in mating success assays. In six independent assays with the FL12-56 line, we found no evidence of cross mating (Fig. 5a). Control experiments, using wild type *C. macellaria* males, resulted in over 80 % of mated females. Likewise, FL12-56 males mated successfully with wild type *C. hominivorax* J06 females under the same experimental conditions.

As the natural populations of *C. hominivorax* in South America and the Caribbean may present some degree of genetic variability, it is worthwhile to investigate the penetrance of the female killing system in different genetic backgrounds (Fig. 5b). We selected a dominant lethal transgenic strain to study penetrance of the female lethal transgene, as this characteristic is of vital importance for a potential future release of fertile transgenic insects in the field. Males of the FL11-18B sexing strain were crossed to wild type females collected from different locations in Central and South America, including Jamaica (J06 and J03), Colombia (Valledupar), Brazil, and Panama (P-95). The offspring of these crosses were reared in diet without tetracycline and adults were sexed and counted. No females were obtained from crosses with Brazil or P-95 strains, as we had observed in J06. However, some females survived from the crosses with J03 and Valledupar strains. The association between sex and strain was significant (*P* < 0.0001). That is, the proportion of female survivors did differ significantly across different genetic backgrounds, suggesting that there is standing genetic variation within screwworms that influenced the degree of dominant female lethality.

As the New World screwworm is a tropical insect, it is not expected to be able to disperse to or survive in colder or dryer climates in South America, particularly during winter. To evaluate the potential for our transgenic strains to thrive at lower temperatures and perhaps colonize new areas of North and South America, we studied the longevity of males from the FL12-56 and FL11-15 transgenic strains and the J06 parental strain at 25 °C and 19 °C in the laboratory (Fig. 5c). These transgenic strains were selected because they hold promise for mass rearing and field release due to their female lethality and fitness characteristics. Results from two independent assays showed that, although males from both J06 and the two transgenic lines live longer at the lower temperature, there was no statistically significant relative difference between the two transgenic lines and J06 at 19 °C (log-rank and Wilcoxon tests on Kaplan–Meier survival curves).

## Discussion

The New World Screwworm Eradication Program is a landmark for SIT, as it was the first coordinated effort that used this technique to successfully eradicate an insect pest on a continental scale. From the outset of this program almost 60 years ago, scientists recognized the benefits of a male-only strain for population suppression of insects in the field [20, 21]. However, a sexing strain of this insect was not available and the Screwworm Eradication Program continued to release both males and females in their field operations. In this study, we have developed the first *C. hominivorax* sexing strains, which carry a repressible female-specific lethal transgenic system [11]. In addition to producing only males when reared on diet without tetracycline, the results from a battery of fitness tests suggest that some of the transgenic sexing strains could replace the J06 parental wild type strain currently used for the SIT program in Panama. The majority of the transgenic lines were comparable across several fitness parameters, such as development from eggs to pupae, egg production, and average pupae weight, to the wild type line currently in mass production. Further, three of the most promising lines were comparable to J06 in male mating success and male competition, suggesting they would be sexually competitive in the field with wild type males.

Our findings have several direct benefits to the existing SIT program for the control of the New World Screwworm in Panama. Firstly, by removing females, the costs of rearing would be reduced since fewer insects would be required to maintain the barrier zone in the Panama-Colombia border. Indeed, releasing only males is expected to improve the efficiency of population suppression in the field compared to a bisexual release [8], thus reducing the number of insects required for regular field releases. Secondly, lower doses of irradiation are required to sterilize males compared to females [22]. Since the output from implementation of an efficient FL11 or FL12 line into the production facility would consist of only males, lower irradiation doses could be used. Exposure of the mass reared *C. hominivorax* to lower doses of radiation would improve the fitness of the released males [23]. An estimation of the weekly costs of producing 16 million sterile insects for the regular operations of the COPEG mass rearing facility is shown in Additional file 4. Production costs represent about 20 % of the total costs of the New World screwworm control program. Current weekly production costs for the J06 strain are compared to the potential use of the FL12-56 strain, a dominant lethal male-only strain and an embryonic lethal male-only strain. Assuming that the increased efficiency of population suppression of FL12-56 males is at least twice that of J06 males and females, a reduction of four times the number of insects required for field release is achieved with important savings in diet, consumable, and operational costs. In total, weekly production costs are reduced by half (Additional file 4). Further, a space four times smaller would be enough to mass-rear the number of insects required to maintain the barrier zone in the Panama–Colombia border. Further savings could be introduced by a dominant lethal strain that would not require irradiation and an embryonic lethal strain would allow for greater savings in larval diet. With these strains it may be possible to release fewer males than indicated, if omitting the radiation treatment does lead to a significant increase in fitness. Dispersal costs are not expected to change with any of the strains as the routes flown and planes used for dispersal would not change. The calculations offered here are hypothetical and based on the behavior of the transgenic sexing strains in the laboratory. Each premise and assumption presented will be tested by mass rearing and performance in the field and validated in the future to determine actual savings. While these efficiency gains have been calculated for maintenance of the barrier zone, they would also be applicable to any future eradication program. Thus, the strains developed in this study could make it possible to extend the program to regions where this insect remains a pest.

On a regional level, the New World screwworm remains endemic in South America and some of the Caribbean islands, notably Cuba and Jamaica. Although SIT has been considered for eradication of screwworm in these regions [24], particularly the Caribbean islands and along the West coast of South America, the costs of developing an eradication program in these regions has prevented implementation. The development of sexing strains and the prospect of more efficient population suppression from male-only releases would reduce the costs of an SIT program for these areas, making this technique more accessible to developing countries. Indeed, in order to undertake the eradication of screwworms in a large Caribbean island such as Cuba, the mass production of insects required at the COPEG facility is estimated at approximately 50 million insects per week. The introduction of a male-only transgenic strain could reduce this number of insects by at least four times, considerably reducing the costs of mass-rearing at this scale. Further, the higher efficiency of field population suppression derived from a male-only strain could also increase the relative maximal production capacity of the mass-rearing facility, which is currently estimated at 100 million insects per week. If a male-only strain were released in the field at a scale that is saturating, eradication of the insect could potentially be achieved in less time, leading to substantial savings in dispersal and field operation costs. Most of the transgenic lines evaluated show high penetrance of the female lethal gene construct, producing very low numbers of heterozygous females when reared in diet without tetracycline. Further, in the FL11-15 and FL11-18B lines, this effect is fully dominant making them potentially attractive candidates for a fertile male release, as their fitness and behavioral characteristics are similar to the J06 wild type strain used for the New World Screwworm Eradication Program in Panama. Sterilization of these strains would not be needed for population suppression, as fertile transgenic males would pass on the female-lethal gene to their offspring, which would also be a male-only population. A strain carrying multiple dominant female lethal genes on different chromosomes is predicted to be particularly efficient for population suppression [25]. Potentially fewer flies of a fertile dominant lethal strain may be needed for an eradication campaign compared to a sterile male-only release due to increased fitness of the non-irradiated males. Furthermore, avoiding the use of irradiation would introduce savings in irradiator maintenance and replacement and would eliminate the need of a highly regulated irradiation department (Additional file 4). One consideration for releasing fertile transgenic *C. hominivorax* males is that tetracycline or its derivatives are used to treat infected wounds and can be added as a supplement to feed for livestock. Consequently, there is the potential for female offspring of released males to survive. Although there is little published information on the level of tetracycline in plasma of cattle in Panama, the maximum residue limits for tetracycline, oxytetracycline, or chlortetracycline in cattle liver and muscle are 600 and 200 μg/kg, respectively [26]. This is more than an order of a magnitude below the lowest tetracycline dose we tested (3 μg/mL), which was not sufficient for female survival. Thus, it would appear unlikely that the female offspring of a dominant lethal strain would survive in the field. Therefore, the economic savings involved in avoiding the use of an irradiator for the production routine of a SIT program, coupled with eliminating the potential health risk to employees, would allow for smaller, healthier, and less costly regional mass facilities to be built, which could promote the development of an eradication program for New World screwworm in South America and the Caribbean.

Although transgenic sexing strains carrying the auto-regulated tTA system have been developed for other insects of economic importance [10, 13, 27, 28], our study is unique in that it was completely performed within the New World screwworm biosecure facility in Panama, where the research focus was to develop a sexing strain that would be directly applied to their regular operations. Indeed, the strains were evaluated for fitness parameters commonly used within the production facility as quality controls to assess a strain for mass rearing. The sexing strains of *C. hominivorax* showed high productivity in all of these fitness parameters as well as a high degree of competitiveness in sexual behavior assays, suggesting that the most promising strains would perform well in mass rearing and be competitive in field trials with wild type males. Moreover, although several research laboratories have created transgenic sexing strains in various pest insects, none of these sexing strains have been incorporated yet into an operational SIT or fertile-release program. Currently, the only open field trials using transgenic insects have been conducted with an *Ae. aegypti* strain that carries a dominant auto-regulated tTA gene that is not sex-specific [29, 30]. From this perspective, an advantage of our program is that the transgenic male-only strains have been developed within the biosecure *C. hominivorax* mass-rearing facility in Panama, where they have the potential to transition from research and development to mass rearing and, eventually, field testing very effectively because they would be integrated to an ongoing SIT program in which the infrastructure and expertise is already routine.

Another novel aspect of this work is a preliminary risk analysis performed as a first step towards addressing potential environmental concerns of a future field release. We show that transgenic *C. hominivorax* males do not mate with wild type *C. macellaria* females, the most closely related species that share its geographic distribution, suggesting that the hybridization of transgenic *C. hominivorax* to other related species in the field would be highly unlikely. We also performed male longevity assays at different temperatures and found that, although both transgenic and wild type males live longer at lower temperatures, there is no evidence for relative increased survival of transgenic males with respect to J06 wild type males at lower temperatures, which could give them an advantage to establish in cooler climates non-endemic to their actual distribution. Finally, we studied the penetrance of the female lethal system in different genetic backgrounds and found that the FL11-18B strain was 100 % dominant female lethal in some, but not all, genetic backgrounds. This suggests that, in some locations, a small percentage of the female offspring of released transgenic males could survive and reproduce. However, only a small number of females were observed in these crosses, suggesting that population suppression would still be very effective. Indeed, the transgenic *Ae. aegypti* strain that has been evaluated in open field trials [30, 31] is not fully dominant with 3–5 % of offspring surviving when reared on diet without tetracycline [32]. Nevertheless, releases of the transgenic strain have effectively suppressed local populations of *Ae. aegypti* [30, 31]. From an environmental perspective, a potential concern would be that the locus or loci responsible for “rescuing” females could sweep to fixation under a sustained fertile release program, similar to the rapid evolution of resistance in the over application of insecticides [33–35]. Consequently, before any fertile field release, it will be necessary to evaluate screwworm from the target population by crossing with a transgenic strain to determine the degree of heterozygous female lethality. With this knowledge, a viable release program can be designed that maximizes population suppression.

Considering the extensive testing of the sexing strains developed in this study and their promising characteristics, COPEG has chosen to evaluate the FL12-56 transgenic strain under mass-rearing conditions. Further, an application for conducting open field trials with radiation sterilized transgenic males has been submitted to the Panamanian Government. The New World Screwworm Eradication Program was the first and is arguably the most successful biological control program targeted against a specific insect pest. Given the highly successful history of the program, we would think it is appropriate that one of the transgenic lines developed in this study would be among the first to be evaluated in open field testing.

## Conclusions

We have developed the first sexing strains of the New World screwworm within the biosecure mass-rearing facility of the New World Screwworm Program in Panama. The fitness characteristics of several transgenic lines, as well as their mating competitiveness suggest a strong potential for application in the current SIT control program. A preliminary risk analysis also shows promise for the use of the sexing strains in the field. We therefore propose that the development of the FL11 and FL12 strains will help promote a more efficient and sustainable SIT control program against this devastating insect pest.

## Methods

### New World screwworm rearing and germ-line transformation

The J06 wild type strain of *C. hominivorax* was collected in Jamaica in 2006 and is the parental strain that is reared routinely at the COPEG biosecurity plant. Adult females are stimulated to lay eggs by presenting them with warm containers of raw ground meat mixed with an attractant made from spent larval media [36]. Eggs are collected from adult females on the sixth day and are seeded in artificial larval diet (containing dry blood, dry egg, dry fishmeal protein, and cellulose fiber) [36] and kept at 39 °C and 80 % humidity for 3 days, adding more food daily, until they have reached the third instar stage. On the fourth day of development, the larvae are placed in a room at 31 °C and 80 % humidity for pupation into containers of sawdust. On the eighth day, the pupae are sieved out of the sawdust and placed into cages for emergence of adults in a colony room at 25.5 °C and 55 % humidity. Insects were reared in a 12 h/12 h light/dark cycle. The J06 strain was used for *piggyBac*-mediated germ-line transformation using a protocol similar to the one developed for *L. cuprina* [37]. Specifically, pre-blastoderm embryos were injected with a mix of pBac [FL] plasmid (800 ng/μL), Lchsp83-pBac helper plasmid (400 ng/μL), and pBac RNA helper (400 ng/μL). A DNA template was prepared and in vitro synthesis of *piggyBac* RNA helper was performed as described previously [11]. First instars showing transient expression of the ZsGreen or DsRedex2 marker were selected and raised on diet supplemented with 150 μg/mL tetracycline. G_0_ adults were crossed to wild type flies and their offspring were screened for expression of the fluorescent marker as first instars. Homozygous *C. hominivorax* individuals were selected as crawl-off third instars based on fluorescence intensity and bred to create a stable line.

### Female lethality tests

Homozygous lines were tested by setting up a cage with 100 flies with food and water without tetracycline. Females were induced to deposit eggs on the sixth day and eggs were collected and divided into two groups of the same weight. One group was seeded in larval diet containing 150 μg/mL tetracycline and the other group was seeded in diet without tetracycline. Larvae were reared to adults and the number of pupae, males, and females was counted. To measure dominant lethality, 10 homozygous transgenic males were crossed with 20 J06 virgin females in a cage with food and water without tetracycline. Larval offspring were raised as described above and the number of emerged adults counted. Three or more independent tests were performed for each line for both tests of female lethality. For testing the effect of tetracycline concentration on female viability, the strains were also tested on a larval diet that was identical to the standard diet except the dry fishmeal protein was replaced with soy flour [15].

### Fitness tests

General fitness tests were performed for all the transgenic lines and for the J06 parental wild type strain, according to protocols used regularly in the COPEG biosecurity facility for quality control [38]. All tests were replicated at least three times unless otherwise indicated.

### Survival from eggs to pupae

For each transgenic line and for the J06 strain, 75 mg of eggs were seeded in larval diet and raised until the pupal stage. The total volume of pupae was measured using a graduated cylinder and the total number of pupae in 25 mL was counted. From this, the total number of pupae and the average pupal weight was calculated. Regular testing has established that 75 mg of eggs is equal to 1875 eggs, and therefore the percentage of eggs that develop into pupae can be calculated.

### Fertility

To measure the fertility of each line, eggs were collected and the egg mass dissociated into individual eggs by incubating them in a 4 % w/v sodium hydroxide solution for 2 min with constant stirring and then rinsing them with abundant distilled water [39]. For each line, 300 individual eggs were placed in a petri dish containing a damp paper towel and black filter paper on top. The petri dishes were incubated at 37 °C overnight and, the following morning, the number of hatched larvae were counted and the percentage egg hatch was calculated.

### Fecundity

For each transgenic line and the J06 strain, a cage was set up with 50 males and 50 females with food and water containing tetracycline. On the sixth day after emergence, the females were induced to lay eggs and the total weight of the egg masses was measured. The average total number of eggs laid per female was calculated by dividing the total egg weight in mg by the number of females in the cage and then multiplying by 25 as 100 mg of eggs equals approximately 2500 eggs.

### Adult emergence and sex ratio

For each transgenic line and a J06 control, 100 pupae were placed in a closed container and adults were allowed to emerge for 3 days after the emergence of the first insect. Males and females were counted and percentage of emergence and sex ratio calculated.

### Male mating success assays

Transgenic and J06 wild type strains were reared under the same conditions of diet without tetracycline, temperature, and humidity. When adults emerged, males and females were immediately collected and placed in separate cages for 4 days to allow them to attain sexual maturity. On the fourth day, 15 virgin J06 females and five transgenic or five J06 males were placed in a rectangular metal cage (15 cm width, 15 cm height, and 25 cm length) for 8 h and the males were then removed from the cage. The following morning, the spermatecae of the females was dissected and observed with the light microscope to assess the presence of sperm as an indication of mating. The number of mated females was counted and the percentage calculated. This experiment was replicated at least three times for each line.

### Male competitiveness assays

Transgenic and J06 wild type strains were reared under the same conditions of diet without tetracycline, temperature, and humidity. When adults emerged, males and females were immediately collected and placed in separate cages for 4 days to allow them to attain sexual maturity. On the fourth day, 10 transgenic males, 10 J06 males, and 10 J06 virgin females were placed in rectangular metal cages (60 cm height, 300 cm width, 60 cm length) for 20 h. The next day, the males were removed from the cage and the females were left until the sixth day. Females were then placed individually in 50 mL Falcon™ tubes containing a small piece of warm raw meat with attractant at the bottom to induce egg laying. The Falcon™ tubes were put in a 37 °C incubator for 2 h and then the egg masses were collected from each tube. The individual egg masses were placed in small petri dishes containing damp paper towel and a small volume of larval diet, labeled, and incubated at 37 °C until hatching. The first instars of each petri dish were observed with a stereo fluorescence microscope to assess paternity. Fluorescent larvae were consistent with mating of females to transgenic males while non-fluorescent larvae were the result of females mating to J06 males. No mixed populations of larvae were observed in this assay, as the limited time of 20 h given to the insects for mating is enough to prevent re-mating of the females to a second male. These tests were replicated at least three times.

To quantify male sexual competitiveness, the MCI was calculated using the formula from [40]:

$$ MCI=\frac{TW}{TW+WW} $$

Here, the mating of transgenic males to J06 wild type females (*TW*) and the mating of J06 wild type males to J06 wild type females (*WW*) was evaluated and values of MCI varied between 0 and 1, where 0 indicates that all the J06 wild type females mated to J06 wild type males, 1 indicates that they all mated to transgenic males, and 0.5 indicates that half mated with J06 wild type males and half mated with transgenic males, and that transgenic males are equally competitive to J06 wild type males.

### Male longevity at lower temperatures

For the FL12-56 and FL11-15 transgenic lines and for the J06 control, two cages were set up with 50 males. In one cage, the flies were kept at 25 °C and in the other they were kept at 19 °C. The flies were given fresh food every 4 days. Every day the cages were checked for dead flies and these were counted until there were no live flies left in the cages. Two independent assays were performed at both temperatures.

### Interspecific crossing to *C. macellaria*

Transgenic *C. hominivorax* lines and a wild type *C. macellaria* strain (collected in Panama) were reared under the same conditions of diet, temperature, and humidity. When adults emerged, males and females were immediately collected and placed in separate cages for 4 days, to allow them to attain sexual maturity. On the fourth day, five transgenic *C. hominivorax* males and 15 wild type *C. macellaria* virgin females were placed in rectangular metal cages (15 cm width, 15 cm height, and 25 cm length) for 3 days; separate control cages holding five wild type *C. macellaria* males with 15 *C. macellaria* females as well as five transgenic *C. hominivorax* males with 15 J06 *C. hominivorax* females were concurrently used. After this time, the spermatecae of the females was dissected and observed with a light microscope to assess the presence of sperm as an indication of successful mating. For each experiment, three replicates were set up for each line of transgenic males. Three independent experiments were performed.

### Heterozygous female lethality in diverse genetic backgrounds

Crosses were performed using 10 males of the FL11-18B transgenic line and 30 virgin females of the different wild type strains collected in South America and the Caribbean: J03, J06, Brazil, Valledupar, and P-95. Males were removed from the cages on the fifth day and females were induced to lay eggs on the sixth day. The egg masses laid by each cage were weighed and half of the egg mass was seeded in diet containing tetracycline and the other half was seeded in diet without tetracycline. The larvae were fed each day with the corresponding diet and reared to adults. After emergence of all the adults, the number of males and females was counted. A single replicate experiment was performed for each strain.

### Statistical analysis

To test the effect of tetracycline on sex, flies were aggregated for each line (between 3 and 8 bottles, for a total of approximately 500–1000 flies per line, per tetracycline treatment, see Additional files 1 and 2). Fisher’s exact test indicated a highly significant association (*P* values all less than 0.002) between sex and tetracycline, with female proportions equal to or very near 0 for each line. When data are aggregated over lines, the *P* value is < 0.0001. To test the effect of tetracycline level and female viability, a three-factor (strain, diet, tetracycline level) analysis of variance (ANOVA) was used to analyze the percentages of survivors that were female. The ANOVA table, along with an interaction plot of means, suggested that strain and tetracycline dosage level explain almost all of the variability in observed female survivorship. Separate tests of strain effects on survivorship were conducted at each level of tetracycline, both for fixed diet. For the general fitness parameters, six response variables of potential interest were measured on each of three replicate assays per line. Simple one-factor linear models were fit to each and all pairwise comparisons with the control (J06) were carried out using Dunnett’s procedure for control of experiment-wise error rate.

For both male mating success and male competition, the data were analyzed with generalized linear models for observed count variables, which were assumed to follow binomial distributions. These models were fit using the GLIMMIX procedure of the SAS statistical software package. For the mating success data, the overall hypothesis of equal probabilities among all 10 lines was tested. Additionally, all pairwise comparisons among the estimated probabilities for the 10 lines were carried out (with Tukey adjustment for control of experiment-wise error rate). For the competition data, the hypothesis of full competition was tested simply by a statistical comparison of the observed frequency with the hypothetical value of 50 %. Again, a generalized linear model was fit to the mating frequencies using PROC GLIMMIX. Cage/experiment effects were included in the model in order to enable investigation of over-dispersion. In the absence of over-dispersion, mating frequencies were aggregated over cages to conduct the test.

For the analysis of female viability in different genetic backgrounds, Fisher’s exact test was used to test the association between sex and strain in surviving adults from the crosses of FL11-18B with strains from different geographic regions, with between 100 and 600 flies sampled from each region. For analysis of the longevity data, Kaplan–Meier survival curves were estimated for each combination of temperature and strain after combining replicates, so that for each of the eight combinations of temperature and strain, 100 flies were at risk on day 0. At each temperature, Log-rank and Wilcoxon statistics were computed to tests for equality of survivor functions across the four strains. In the evidence of strain effects, subsequent pairwise comparisons were also made, again using log-rank and Wilcoxon tests, with Tukey adjustment for multiplicity of comparisons. Additionally, estimates of the mean and median survival time were computed for each strain and temperature. All of these computations were carried out using the LIFETEST procedure in SAS.

## Additional files

Additional file 1: Female lethality data for *C. hominivorax* FL11 and FL12 homozygous lines. (XLSX 38 kb) Additional file 2: Female lethality data for *C. hominivorax* FL11 and FL12 heterozygous lines. (XLSX 36 kb) Additional file 3: Diet tetracycline concentration and female viability for the FL12-56 and control J06 strains. A tetracycline dose response assay was performed rearing insects in fishmeal protein diet (A) and in soy protein diet (B). Three replicate experiments were performed with 300–400 flies counted for each replicate. Similar results were obtained for both diets, in which females required doses of tetracycline higher than 100 mg/L in order to survive to adults. Mean ± standard deviation are shown. (PDF 225 kb) Additional file 4: Summary of the main current weekly production costs of the New World screwworm mass rearing facility and potential savings derived from the use of male-only strains. The table reflects the production costs of the mass rearing facility for maintaining the barrier at the Panama–Colombia border. Working from the premise that a male-only strain will be at least two times more efficient in field population suppression than a bisexual strain, we reasoned that the number of insects needed for field release would be four times less than the current production. With this assumption, the space required to produce 75 % less insects would be much smaller and savings could be made in larval diet, power and water consumption, maintenance of the irradiators, general equipment (such as insectronics), and the number of personnel required to operate the plant. Further savings can be achieved using a dominant lethal strain, which would eliminate the need for irradiation. An irradiator and source cost 1.5 million US dollars to buy and the source is replaced every 5 to 7 years at a cost of 0.9 million US dollars. The cost of maintenance of the irradiator and replacement of the source is calculated normalized per week over 6 years. An embryonic female lethal strain would introduce further savings in larval diet but would require the same space and people for production in the current facility. The number of insects is expressed in millions of insects released weekly (M). All figures are given in US dollars. Each premise and assumption presented will be field tested and validated in the future to determine actual savings. (DOCX 101 kb)

## Abbreviations

COPEG: Comisión Panamá – Estados Unidos para la Erradicación y Prevención del Gusano Barrenador del Ganado
SIT: Sterile insect technique
*tra*: Transformer
tTA: Tetracycline transactivator
