# Supplementary figures and images for: A transgenic male-only strain of the New World screwworm for an improved control program using the sterile insect technique
Source: BMC Biol. 2016 Aug 30;14(1):72. doi: 10.1186/s12915-016-0296-8 (PMC5004303; doi:10.1186/s12915-016-0296-8)

**A**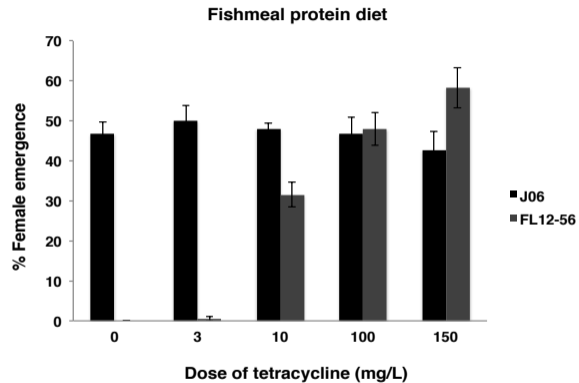**B**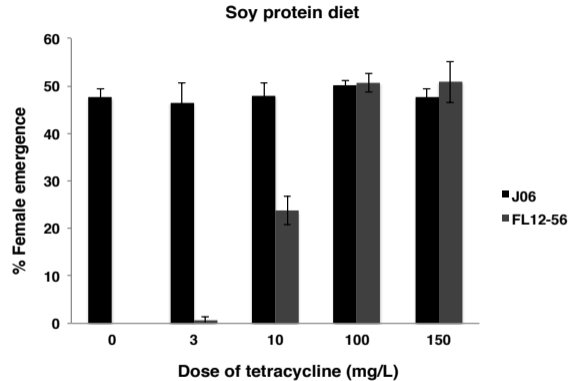

Supplement: Additional file 3: — Diet tetracycline concentration and female viability for the FL12-56 and control J06 strains. A tetracycline dose response assay was performed rearing insects in fishmeal protein diet (A) and in soy protein diet (B). Three replicate experiments were performed with 300–400 flies counted for each replicate. Similar results were obtained for both diets, in which females required doses of tetracycline higher than 100 mg/L in order to survive to adults. Mean ± standard deviation are shown. (PDF 225 kb) [file 12915_2016_296_MOESM3_ESM.pdf]
