# Supplementary material for: A transgenic male-only strain of the New World screwworm for an improved control program using the sterile insect technique
Source: BMC Biol. 2016 Aug 30;14(1):72. doi: 10.1186/s12915-016-0296-8 (PMC5004303; doi:10.1186/s12915-016-0296-8)
Supplement: Additional file 4: — Summary of the main current weekly production costs of the New World screwworm mass rearing facility and potential savings derived from the use of male-only strains. The table reflects the production costs of the mass rearing facility for maintaining the barrier at the Panama–Colombia border. Working from the premise that a male-only strain will be at least two times more efficient in field population suppression than a bisexual strain, we reasoned that the number of insects needed for field release would be four times less than the current production. With this assumption, the space required to produce 75 % less insects would be much smaller and savings could be made in larval diet, power and water consumption, maintenance of the irradiators, general equipment (such as insectronics), and the number of personnel required to operate the plant. Further savings can be achieved using a dominant lethal strain, which would eliminate the need for irradiation. An irradiator and source cost 1.5 million US dollars to buy and the source is replaced every 5 to 7 years at a cost of 0.9 million US dollars. The cost of maintenance of the irradiator and replacement of the source is calculated normalized per week over 6 years. An embryonic female lethal strain would introduce further savings in larval diet but would require the same space and people for production in the current facility. The number of insects is expressed in millions of insects released weekly (M). All figures are given in US dollars. Each premise and assumption presented will be field tested and validated in the future to determine actual savings. (DOCX 101 kb) [file 12915_2016_296_MOESM4_ESM.docx]

| **Weekly Production** | **Current J06 strain** | **FL12-56** | **Dominant lethal strain** | **Dominant embryonic lethal strain** |
| --- | --- | --- | --- | --- |
|  |  |  |  |  |
| % male larvae | 50% | 50% | 50% | 100% |
| Racks with larvae | 42 | 22 | 22 | 12 |
| Cost larval diet | $6853 | $3426 | $3426 | $1868 |
| % male pupae | 50% | 68% | 68% | 100% |
| % adult female flies | 50% | 0% | 0% | 0% |
| Sterile pupae | 18.3 M | 6.35 M | 6.35 M | 4.58 M |
| Sterile Flies | 16.34 M | 4.08 M | 4.08 M | 4.08 M |
| Irradiator replacement and maintenance | $3,691 | $3,288 | $0 | $0 |
| Irradiation department | 15 people | 5 people | 0 people | 0 people |
| Irradiation personnel | $5368 | $1789 | $0 | $0 |
| Space | 4974 m^2^ | 1105 m^2^ | 1105 m^2^ | 1105 m^2^ |
| Power (electricity + diesel) | $6300 | $3560 | $3560 | $3560 |
| Water | $1400 | $840 | $840 | $840 |
| Production department | 50 people | 26 people | 26 people | 26 people |
| Production personnel | $14127 | $7063 | $7063 | $7063 |
| Biosecurity department | 12 people | 5 people | 5 people | 5 people |
| Biosecurity personnel | $4458 | $1857 | $1857 | $1857 |
|  |  |  |  |  |
| Total weekly | $42197 | $21823 | $16746 | $15188 |
